# A Neck of Femur Fracture Associated With Electric Scooter Use in a Young Patient

**DOI:** 10.7759/cureus.69014

**Published:** 2024-09-09

**Authors:** Owen Chu, Qamar Mustafa, Iain Findlay

**Affiliations:** 1 Trauma and Orthopaedics, Dorset County Hospital NHS Foundation Trust, Dorchester, GBR

**Keywords:** electric scooter, e-scooter, e-scooter injury, neck femur fracture, orthopaedics trauma, traumatic hip fracture, young adult male

## Abstract

Since the launch of electric scooter (e-scooter) trials in the UK by the Department for Transport (DfT), there has been an upward trend in injuries involving e-scooters. We report a case of a significant orthopaedic injury in a male patient following a fall from an e-scooter. He sustained a right-sided neck of femur fracture. He was treated successfully with a dynamic hip screw. Hip fracture in the young population is rare and is often associated with higher complication rates. We emphasise the importance of prompt identification and operative management of traumatic hip fractures in young patients and raising safety awareness about the use of e-scooters.

## Introduction

The use of electric scooters (e-scooters) has become increasingly popular across the world ever since the introduction of hired electric scooters. Since the launch of e-scooter trials in the UK by the Department for Transport (DfT), there has been an upswing in injuries involving e-scooters. According to national statistics, there were 1402 e-scooter accidents in 2022, up from 882 in 2021 [1]. Similar trends were observed across Europe and in major cities around the world where e-scooters were introduced. In France, the French National Trauma Registry recorded an 184% increase in road traffic accidents (RTAs) involving e-scooters between 2019 and 2022 [2].

Several studies have reported a growing concern about the rise in e-scooter injuries. Findings suggest that the head and upper extremities are the two most common regions affected by e-scooter injuries [3]. Recent studies have shown a significant proportion (9%) of e-scooter-related hip fractures in young adults and e-scooters were the leading cause of traumatic hip fractures in the young [4,5]. Although the common e-scooter-related injuries were usually reported to involve the upper limbs, these data demonstrated that significant injuries like hip fractures can also be associated with e-scooters. We report a case of a significant orthopaedic injury in a male patient following a fall from an e-scooter. He was treated successfully with a dynamic hip screw. Hip fracture in the young population is rare and associated with higher complication rates.

## Case presentation

**Figure 1:**
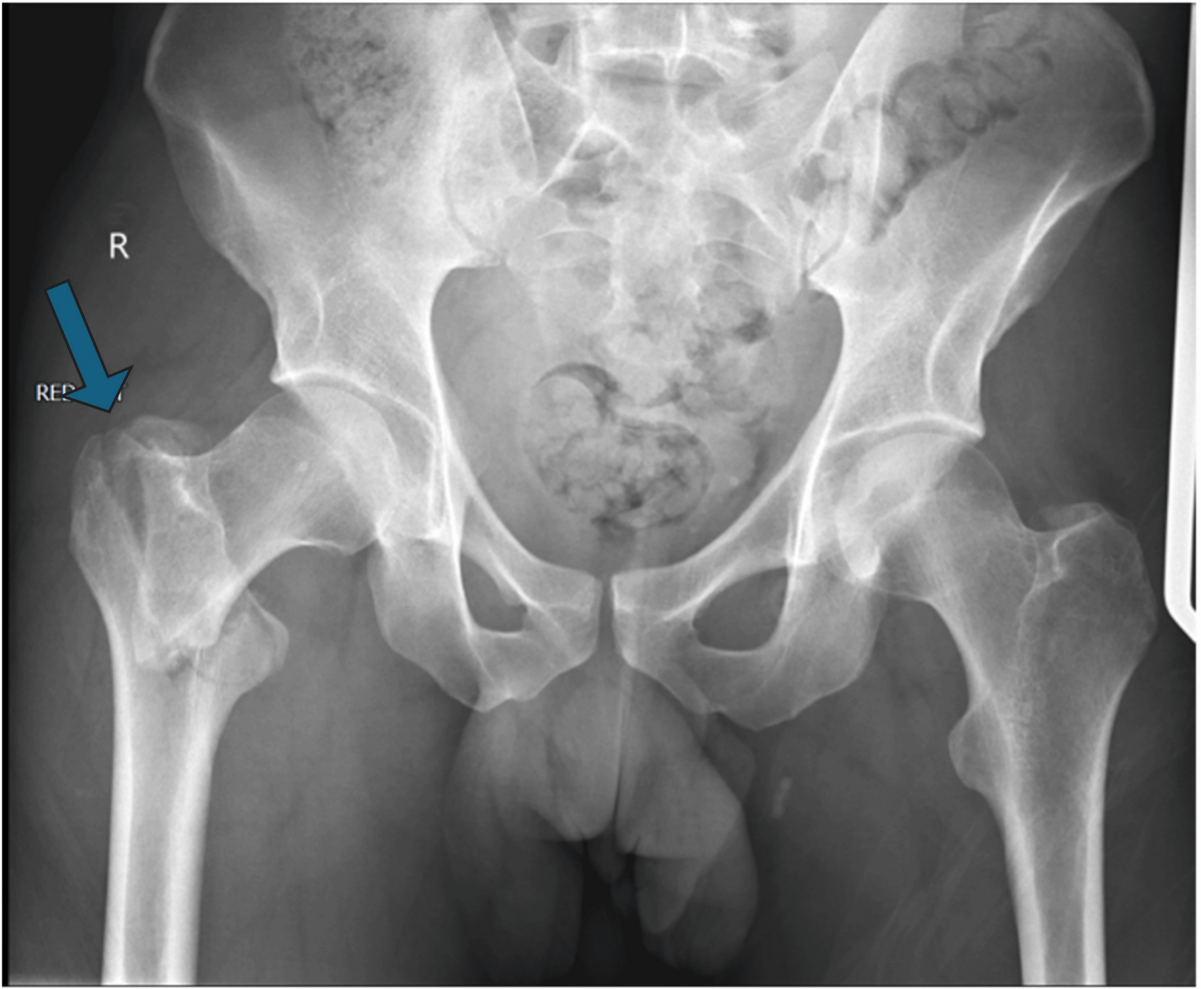
Plain radiograph of the pelvis showing a displaced right sided inter-trochanteric neck of femur fracture (arrow)

A male in his early 20s was brought to the Emergency Department after falling off his e-scooter. He had driven onto the curb at approximately 16 miles per hour and fallen onto his right-hand side. He reported severe pain in his right hip and was unable to weight-bear immediately after the injury. Physical examination demonstrated a shortened and externally rotated right leg with bruises over the greater trochanter. Tenderness was elicited on axial compression and log-rolling of the hip. The patient was unable to straight leg-raise. Peripheral pulses of the right foot were present with good volume. The sciatic nerve was functioning with preserved sensory and motor function in its peripheral distribution. A plain radiograph of the pelvis showed a displaced right inter-trochanteric neck of femur fracture (Figure 1).

The patient was cardiovascularly stable on arrival with a systolic blood pressure of 133 mmHg and a heart rate of 72 beats per minute. He reported having a pain score of 8/10 [6]. He was given 400 mg ibuprofen, 1 g paracetamol, Entonox and 10mg IV morphine. A full set of blood was taken as per the fractured neck of the femur integrated care protocol. A fascia iliaca block was performed with 40 mg levobupivacaine by the emergency team to provide adequate analgesia. The patient was referred to the on-call orthopaedic team following initial resuscitation. He denied any significant past medical history and alcohol use. He was normally fit and well, independent and a smoker (10-pack year history). He was advised to be nil-by-mouth.

The patient was reviewed by the on-call registrar and, following a discussion of options, he consented to the fixation of the right hip fracture. Particular attention was paid to the discussion of complications, which include avascular necrosis, delayed union, non-union, implant failure and reoperation. The case was added to the trauma theatre list. Following discussion at the ‘Orthopaedic Trauma Meeting’ on the same day, he was reviewed by the post-take consultant and consent was confirmed. He was sent to the trauma theatre as the first patient on the list.

Preoperative planning

A dynamic hip screw device was selected as the fracture pattern demonstrated an intact lateral wall, a stable pattern with an intact lesser trochanter. Following the evaluation of the angle between the femoral neck and the shaft of the contralateral femur, a 135 ° barrel angle was selected. We anticipated the patient to have a thick, soft tissue envelope and hard bone.

Surgical procedure

The patient was positioned supine on the operating table with his left uninjured leg flexed and abducted and placed in a gutter. The injured leg was placed into traction on a radiolucent table. Closed reduction was achieved by pulling in the direction of the long axis of the leg to distract the fragments and regain length, and internal rotation of the femoral shaft, with the reduction confirmed under image intensification. Following routine preparation and draping, a longitudinal incision was made 1cm proximal to the lesser trochanter, extending distally approximately 5 cm. The fascia lata was split inline of its fibres, exposing the vastus lateralis. To mitigate damage to the vastus lateralis, a subvastus approach was utilised and the muscle was reflected using 2 ring handle spikes. Prompt haemostasis was utilised using monopolar diathermy and the proximal lateral femoral shaft was exposed. 

**Figure 2:**
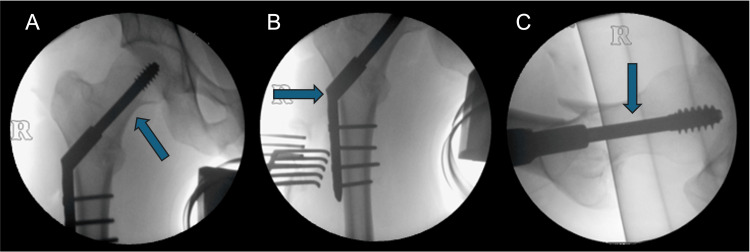
Intraoperative fluoroscopy with a dynamic hip screw fixation in situ (A,B) Anteroposterior and (C) lateral fluoroscopic view demonstrating dynamic hip screw in situ (arrows)

A K-wire was inserted through the dynamic hip screw angle guide (135 °) and placed on the femoral shaft. To avoid rotational displacement during screw insertion, an antirotation wire was placed anterior and parallel to the head-neck axis. A guide wire was then inserted into the centre of the femoral head, parallel to the K-wire. Insertion depth was measured at 110 mm. The dynamic hip screw triple reamer was adjusted to the chosen length and was placed over the guide wire for reaming to a depth of 100 mm. Depth of reaming was monitored with intraoperative fluoroscopy, including checking for advancement of the wire. The channel was tapped to clear the bone. The plate was slid over the guide wire and inserted with a screw. The plate was then fixed to the femoral shaft with four cortical screws. Fracture stabilisation and the position of the dynamic hip screw were confirmed on anteroposterior and lateral views (Figures 2A-2C). All incisions were closed in a layered fashion. The surgical incision was closed with skin clips. 

Postoperative care

Postoperative care was facilitated using a multidisciplinary approach. Regular observations were performed by the nursing team. Daily medical reviews were undertaken to monitor wound, neurological and vascular status. The patient was prescribed enoxaparin 40 mg once a day as per local venous thromboembolism (VTE) prophylaxis protocol. He was also assessed by the therapy team daily to encourage early mobilisation. The patient progressed well and was able to mobilise with crutches by day three post-operation. As such, he was discharged three days after the procedure with a follow-up at the fracture clinic in six weeks. He was given enoxaparin 40 mg as VTE prophylaxis for four weeks and was advised to be fully weight-bearing with crutches for six weeks. Skin clips were removed in the community by the primary care team.

**Figure 3:**
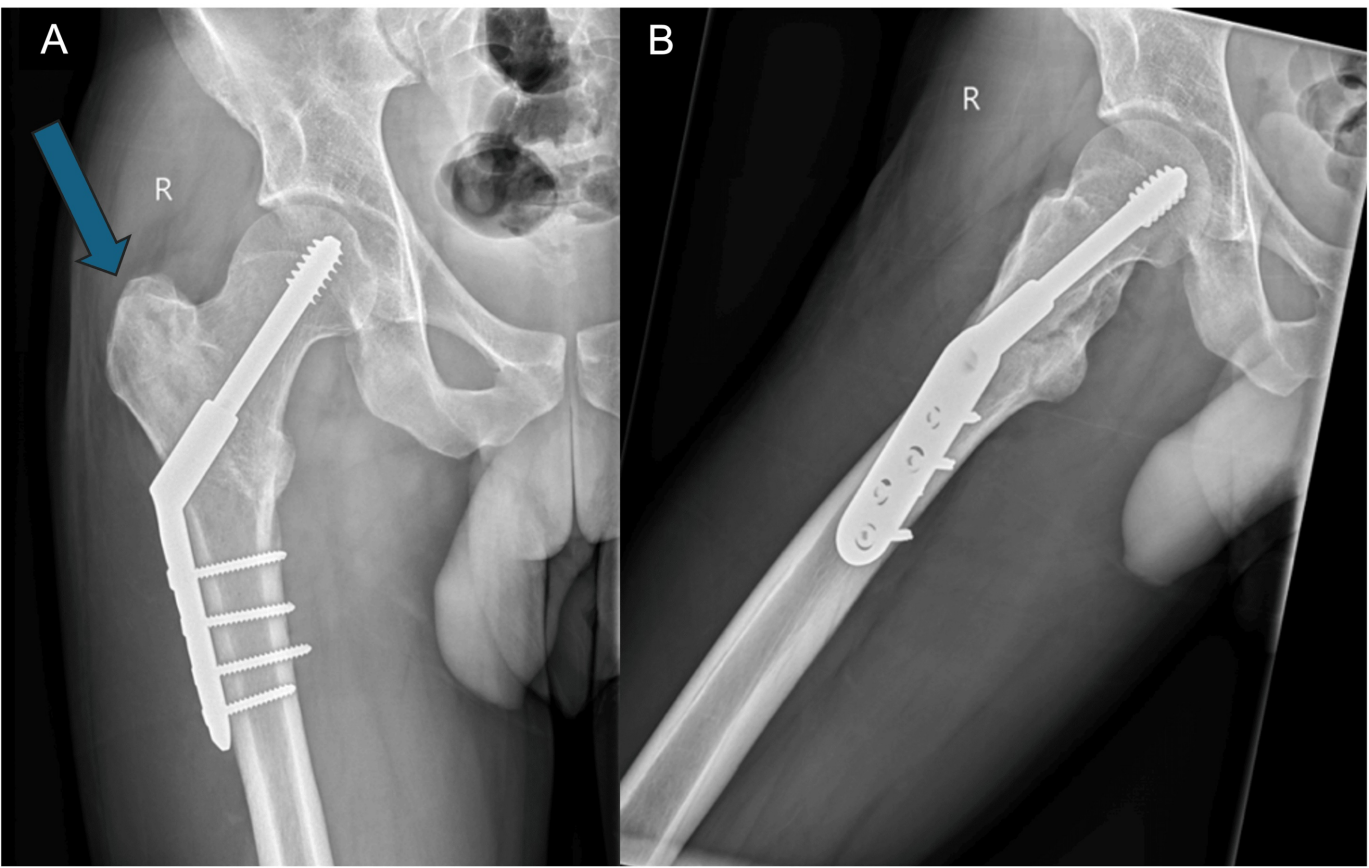
Postoperative radiographs of the right hip at six weeks (A) Anteroposterior view and (B) lateral view demonstrating position of dynamic hip screw and union of fracture (arrow)

He was reviewed at the fracture clinic after six weeks. Plain radiographs demonstrated the satisfactory position of dynamic hip screw and union of fracture (Figures 3A-3B). Clinically, he progressed well with mild pain that was amendable to simple analgesia. There was no complication regarding wound healing. He was requested to appear for a follow-up at one year following a telephone consultation 12 weeks after the procedure.

## Discussion

E-scooters have become a popular means of transport across the world. With its increased usage due to the expansion of hiring schemes and private purchases of e-scooters, e-scooter-related injuries have been on the rise.

Injury pattern

Upper extremity fractures are usually the most common orthopaedic injuries sustained from e-scooters [7-9]. This is consistent with studies in the UK; Bentham et al. have reported a marked increase in radiological investigations and injuries related to e-scooters since the launch of the e-scooter sharing scheme, with upper limb injuries (58%) being the most common type [10]. Due to the design of e-scooters, users would fall from a standing height. They postulated that if users are travelling at high speed, they will use an outstretched arm to break their fall. However, lower limb injuries should not be neglected. Two previous case series reported that about 10% of their patients sustained hip fractures [4,5].

Traumatic hip fracture in young adults

Traumatic hip fractures in young adults (aged <50 years) are generally rare. The most common cause of hip fracture in this age group is high-energy trauma. Two series reported 14 cases of e-scooter-related hip fractures [5]. The seven patients assessed by Ishmael et al. had an age range of 28-68 years, five of whom were aged ≤46 years. This demonstrated that although e-scooter-related hip fractures are uncommon, they do occur. Kayaalp et al. conducted an aetiological analysis of hip fractures in young patients between the ages of 16 and 40 years over seven years between 2016 to 2022. They reported that e-scooters have gone from the fifth most common factor related to injuries in 2016-2020 to the leading factor in 2021-2022 [4]. While there is no other previous study analysing details of traumatic hip fractures in young adults, these data suggest that e-scooter injuries could be devastating. 

Anticipating and planning for problems

Surgical treatment for hip fractures in young adults is not without risk. The incidence of complications experienced by the young neck of femur fracture patients is comparatively high - partly due to the high-energy mechanism as explained above and the displaced fracture patterns. A meta-analysis performed by Slobogean et al. reported reoperation following internal fixation of isolated femoral neck fractures in nearly 20% of cases, and avascular necrosis (AVN) and non-union were the most common complications that likely contributed to repeat surgeries [11]. Treatment outcomes are likely to be affected by several factors, such as the accuracy of reduction and the location of internal fixtures. Therefore, any approach that may reduce the risk of complications is of paramount importance.

Traumatic femoral neck fractures require prompt identification and operative management to allow for fracture reduction, stabilisation, and ultimately functional recovery. Strategies that may reduce the risk of complications in younger patients include satisfactory reduction of the fracture, use of an antirotation wire, placement of the guidewire in the head-neck axis, and tapping for the screw to reduce rotation displacement. Based on the fracture pattern, a lateral approach was favoured as it allows direct fracture visualisation and the application of a subvastus approach. A dynamic hip screw was preferred over an intramedullary nail (IMN) as the latter could violate the abductor muscles of the hip [12]. Not only is a dynamic hip screw less violating, but it also reduces the need for assistance in theatre. Nonetheless, with a subvastus approach, prominent perforating blood vessels may be encountered, which if cut, can retract medially and continue bleeding [13]. In our case, all the aforementioned strategies were employed to minimise the risk of complications.

Regulation of e-scooters

Several studies have looked at the possible factors leading to an increasing rate of accidents related to e-scooters. In the UK, the use of e-scooters remains illegal in public areas unless they are hired through a share scheme. E-scooters in trial schemes are limited to a maximum speed of 15.5 mph and can only be operated by users with a valid full or provisional driving license [14]. However, most of the e-scooter-related injuries were reported to involve private e-scooters than hired e-scooters. Many of these private e-scooters have a higher speed limit, some reaching 30 mph [8]. These likely translate to higher-energy trauma in collisions, e.g., hip fractures.

## Conclusions

The case report highlights that significant lower limb injuries like neck of femur fractures could occur following e-scooter use in young individuals. It emphasises the importance of prompt identification and operative management of traumatic hip fractures in young patients. It also underscores the need for raising safety awareness regarding the use of e-scooters.
